# Analysis of *CDKN1C *in fetal growth restriction and pregnancy loss

**DOI:** 10.12688/f1000research.15016.2

**Published:** 2020-04-21

**Authors:** Jenifer P. Suntharalingham, Miho Ishida, Federica Buonocore, Ignacio del Valle, Nita Solanky, Charalambos Demetriou, Lesley Regan, Gudrun E. Moore, John C. Achermann

**Affiliations:** 1 Genetics and Genomic Medicine, UCL Great Ormond Street Institute of Child Health, University College London, London, WC1N 1EH, UK; 2 Obstetrics and Gynaecology Department, St Mary's Hospital, Imperial College London, London, W2 1NY, UK

**Keywords:** CDKN1C, intra-uterine growth restriction, fetal growth restriction, Silver-Russell syndrome, IMAGe syndrome, adrenal, placenta, recurrent miscarriage

## Abstract

**Background:** Cyclin-dependent kinase inhibitor 1C (CDKN1C) is a key negative regulator of cell growth encoded by a paternally imprinted/maternally expressed gene in humans. Loss-of-function variants in *CDKN1C* are associated with an overgrowth condition (Beckwith-Wiedemann Syndrome) whereas “gain-of-function” variants in *CDKN1C *that increase protein stability cause growth restriction as part of IMAGe syndrome ( Intrauterine growth restriction, Metaphyseal dysplasia, Adrenal hypoplasia and Genital anomalies). As three families have been reported with *CDKN1C* mutations who have fetal growth restriction (FGR)/Silver-Russell syndrome (SRS) *without* adrenal insufficiency, we investigated whether pathogenic variants in *CDKN1C* could be associated with isolated growth restriction or recurrent loss of pregnancy.

**Methods:** Analysis of published literature was undertaken to review the localisation of variants in *CDKN1C* associated with IMAGe syndrome or fetal growth restriction. *CDKN1C* expression in different tissues was analysed in available RNA-Seq data (Human Protein Atlas). Targeted sequencing was used to investigate the critical region of *CDKN1C* for potential pathogenic variants in SRS (n=66), FGR (n=37), DNA from spontaneous loss of pregnancy (n= 22) and women with recurrent miscarriages (n=78) (total n=203).

**Results:** All published single nucleotide variants associated with IMAGe syndrome are located in a highly-conserved “hot-spot” within the PCNA-binding domain of CDKN1C between codons 272-279. Variants associated with familial growth restriction but normal adrenal function currently affect codons 279 and 281. *CDKN1C* is highly expressed in the placenta compared to adult tissues, which may contribute to the FGR phenotype and supports a role in pregnancy maintenance. In the patient cohorts studied no pathogenic variants were identified in the PCNA-binding domain of CDKN1C.

**Conclusion:** CDKN1C is a key negative regulator of growth. Variants in a very localised “hot-spot” cause growth restriction, with or without adrenal insufficiency. However, pathogenic variants in this region are not a common cause of isolated fetal growth restriction phenotypes or loss-of-pregnancy/recurrent miscarriages.

## Introduction

Cyclin-dependent kinase inhibitor 1C (CDKN1C, also known as P57/kip2) (OMIM 600856) is a key negative regulator of cell proliferation that is encoded by a paternally imprinted (maternally expressed) gene on the short arm of chromosome 11 (11p15.4) in humans ( Stampone *et al.*, 2018).

Consistent with its role in growth and development, maternally-inherited *loss-of-function* variants in CDKN1C are found in approximately 5–10% of individuals with the “overgrowth” condition, Beckwith-Wiedemann Syndrome (BWS) (OMIM 130650) ( Eggermann *et al.*, 2014). Clinical features of BWS include macrosomia, hyperinsulinism and adrenal tumors.

In contrast, *gain-of-function* variants in CDKN1C have been shown to cause growth restriction as part of IMAGe syndrome (OMIM 614732) ( Arboleda *et al.*, 2012). IMAGe syndrome is characterised by fetal/ ***I***ntrauterine growth restriction, ***M***etaphyseal dysplasia, ***A***drenal hypoplasia and ***Ge***nital anomalies (in males, usually relatively mild hypospadias and undescended testes) as well as additional features such as hearing loss and hypercalciuria ( Bennett *et al.*, 1993; Vilain *et al.*, 1999).

To date, children with IMAGe syndrome have all been found to harbour pathogenic single nucleotide variants (SNVs) in a very specific region of the PCNA-binding domain of CDKN1C ( Arboleda *et al.*, 2012; Cabrera-Salcedo *et al.*, 2017). These changes potentially lead to increased activity through increasing protein stability, thereby preventing cell cycle progression into S phase ( Borges *et al.*, 2015; Hamajima *et al.*, 2013).

More recently, SNVs in the PCNA-binding domain of CDKN1C have been reported in families with maternally-inherited fetal growth restriction (FGR) *without* adrenal insufficiency and in familial Silver-Russell syndrome (SRS) (OMIM 180860) ( Brioude *et al.*, 2013; Kerns *et al.*, 2014; Sabir *et al.*, 2019). SRS is characterised by variable clinical features including fetal and post-natal growth restriction, relative macrocephaly, feeding difficulties and characteristic facies. SRS is also described as phenotypically and genotypically opposite to BWS and approximately half of the molecular anomalies are attributed to Chr11p15.5 imprinting clusters, including several individuals with maternal duplication of the locus containing CDKN1C ( Bonaldi *et al.*, 2011; Boonen *et al.*, 2016; Schönherr *et al.*, 2007). These findings suggest that the growth restriction phenotype associated with CDKN1C may be more variable and adrenal insufficiency is not always present.

The aim of this study was therefore to review published *CDKN1C* variants associated with FGR/IUGR phenotypes, to study *CDKN1C* expression in different tissues, and to analyse the critical region in CDKN1C in a range of growth restriction and adverse pregnancy phenotypes, with a hypothesis that severe restriction of feto-placental growth may, in some situations, result in pregnancy loss or recurrent miscarriage.

## Methods

### Review of pathogenic SNVs and population variability

A PubMed search was undertaken (March 2020) using the search terms “CDKN1C” with “human” and “growth”, or “IMAGe syndrome”, “IUGR”, “SGA” and “FGR”. Reports focusing on growth restriction phenotypes associated with single nucleotide variants were considered. Population variation in *CDKN1C* was assessed using the gnomAD browser ( http://gnomad.broadinstitute.org; accessed April 2020) ( Lek *et al.*, 2016). Protein conservancy analysis was performed using ClustalW in Jalview ( Waterhouse *et al.*, 2009).

### Analysis of CDKN1C expression

RNA-Seq data for CDKN1C expression was obtained with specific permission from the Human Protein Atlas (Human Protein Atlas available from www.proteinatlas.org) and re-drawn in R (version 3.4.2) ( Uhlen *et al.*, 2015).

### Study cohorts

The following growth restriction cohorts were included in this study: 1) SRS (n=66) (isolated, non-familial) diagnosed on consensus criteria and where maternal uniparental disomy or H19/IGF2:IG-DMR (also known as ICR1 or IC) hypomethylation had been excluded ( Wakeling *et al.*, 2017); 2) FGR (n=37) (isolated, non-familial) defined as birth weight less than the 3rd centile, as part of the Baby Bio Bank cohort (UCL-GOS Institute of Child Health & St Mary’s Imperial College London) ( Leon *et al.*, 2016). Additional analysis was undertaken in DNA from 3) products of conception (POC) (n=22) where there had been a spontaneous loss of pregnancy and 4) women who had a history of recurrent miscarriages (n=78) (at least three miscarriages) where an underlying cause was not known (Baby Bio Bank). An overview of these cohorts is provided in Table 1.

**Table 1.** Overview of the cohorts studied.

Cohort	Number	Characteristics	Main Sequencing Approach
Silver-Russell Syndrome	66 ^a^	Silver-Russell syndrome; maternal uniparental disomy of chromosome 7 or H19/IGF2:IG-DMR hypomethylation excluded	Sanger
IUGR/FGR	37 ^b^	DNA from children with intra-uterine growth restriction (birth weight < 3 ^rd^ percentile) (Baby Bio Bank)	Next-generation sequencing (HaloPlex HS mean read depth 37.8)
Products of conception	22	DNA from lost products of conception between 9–11 weeks gestation	Next-generation sequencing (HaloPlex HS mean read depth 58.5)
Recurrent miscarriages	78	DNA from mothers with recurrent miscarriages (>3) and usually a history of live births	Next-generation sequencing (HaloPlex HS mean read depth 31.3)

Abbreviations: FGR, fetal growth restriction; IUGR, intrauterine growth restriction. ^a^includes an additional 8 children sequenced using a Nonacus Cell3 ^TM^ Target panel (median read depth =4000, range 118–6750); ^b^includes an additional 11 children sequenced using a Nonacus Cell3 ^TM^ Target panel (median read depth 5950, range 20–10837).

### Consent

Ethical Committee approval for the Baby Bio bank was obtained from the Trent Derby Ethics Committee (09/H0405/30) and Ethical Committee approval for the Silver Russell trios was from GOSH Research Ethics Committee (REC No. 1278). Written informed consent was obtained from participants or parents. DNA was extracted from blood lymphocytes, placental tissue or products of conception, as appropriate.

### Genomic analysis of CDKN1C by Sanger Sequencing

Direct Sanger Sequencing was undertaken for 58 SRS patients to analyse the PCNA-binding region (codons 213–316) and hotspot (codons 272–281) using primers reported previously (CCDS7738, ENST00000414822.8) ( Arboleda *et al.*, 2012). Additional primers pairs were used to sequence the 3’ end of exon 1 and splice site (CDKN1CF: CAGGAGCCTCTCGCTGAC; CDKN1CR2: GCTGGAGGGCACAACAAC). Polymerase chain reaction (PCR) was carried out with BIOTAQ DNA Polymerase (BIOLINE, London, UK). PCR products were purified by microclean (Microzone, Haywards Heath, UK) and amplified with BigDyeTerminator v1.1, followed by sequencing on a DNA Analyzer 3070 (Applied Biosystems, California, US). The resulting read-outs were reviewed in Sequencher (v5.3: Gene Codes).

### Genomic analysis of CDKN1C by targeted capture array and next generation sequencing

Targeted array capture followed by next-generation sequencing was performed for FGR samples, products of conception and mothers with a history of recurrent miscarriage.

A targeted enrichment custom HaloPlex HS panel (501kb–2.5Mb) (Agilent Technologies Inc.) was designed using Agilent SureDesign to capture known and candidate genes for fetal growth disruption, including *CDKN1C*. This study used designs targeting either 147 (design size 1.391 Mbp) or 257 (design size 2.045 Mbp) genes.

Sequencing libraries were prepared using 50ng of genomic DNA following the manufacturer’s protocol (HaloPlex HS Target Enrichment System for Illumina Sequencing version C1 from December 2016) and as described in principle previously ( Guran *et al.*, 2016). This was followed by 2 x 100bp or 2 x 149bp paired end sequencing to a median read depth of 300x on a NextSeq sequencer (Illumina Inc.). The bcl files were converted to fastq files using manufacturers recommended guidelines and were then analysed in SureCall software (version 4.0.1.46 (Agilent Technologies) using the HaloPlex Default Method or custom settings (minimum number of read pairs per barcode =1). Samples with a minimum read depth less than 4 at any single nucleotide position were excluded.

An additional 19 patient samples (8 SRS, 11 FGR) were deep-sequenced using a custom Nonacus Cell3 ^TM^ Target enrichment panel for NGS (Nonacus, Birmingham, UK), using the Manufacturer’s instructions and a protocol described previously ( Buonocore *et al.*, 2019). The panel design captured the coding regions of growth related genes *CDKN1C* and *SAMD9* (Tier 1 design, 1Kb – 1.2Mb, Total target size 7386bp). In brief, genomic DNA samples (100ng) underwent enzymatic shearing followed by end repair and dA tailing to ligate molecular identifier adapters. DNA was purified using Agencourt AMPure beads (Beckman Coulter Inc., USA) to remove nonligated adapters, and amplified using adapter primers. Libraries were hybridised with the customised probes, washed, and then targeted library DNA sequences were amplified. After washing and quantification, the libraries were sequenced on a MiSeq platform (Illumina Inc.). Fastq files were analysed on a bioinformatics pipeline provided by Nonacus and variant calling undertaken in Platypus (v 0.8.1). Inspection of BAM files was done in the Integrative Genomics Viewer. 

## Results

### Single nucleotide variants in CDKN1C

**Figure 1.**
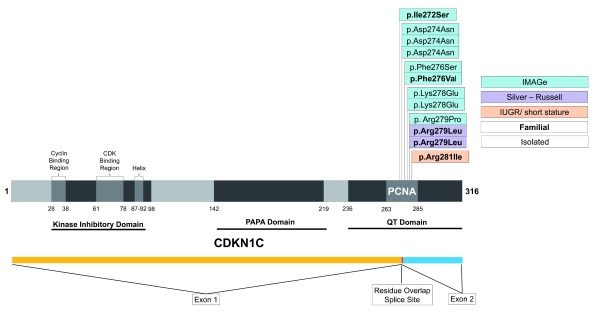
Schematic diagram showing the structure of CDKN1C and the clustering of pathogenic variants associated with IMAGe syndrome and/or growth restriction.

**Figure 2.**
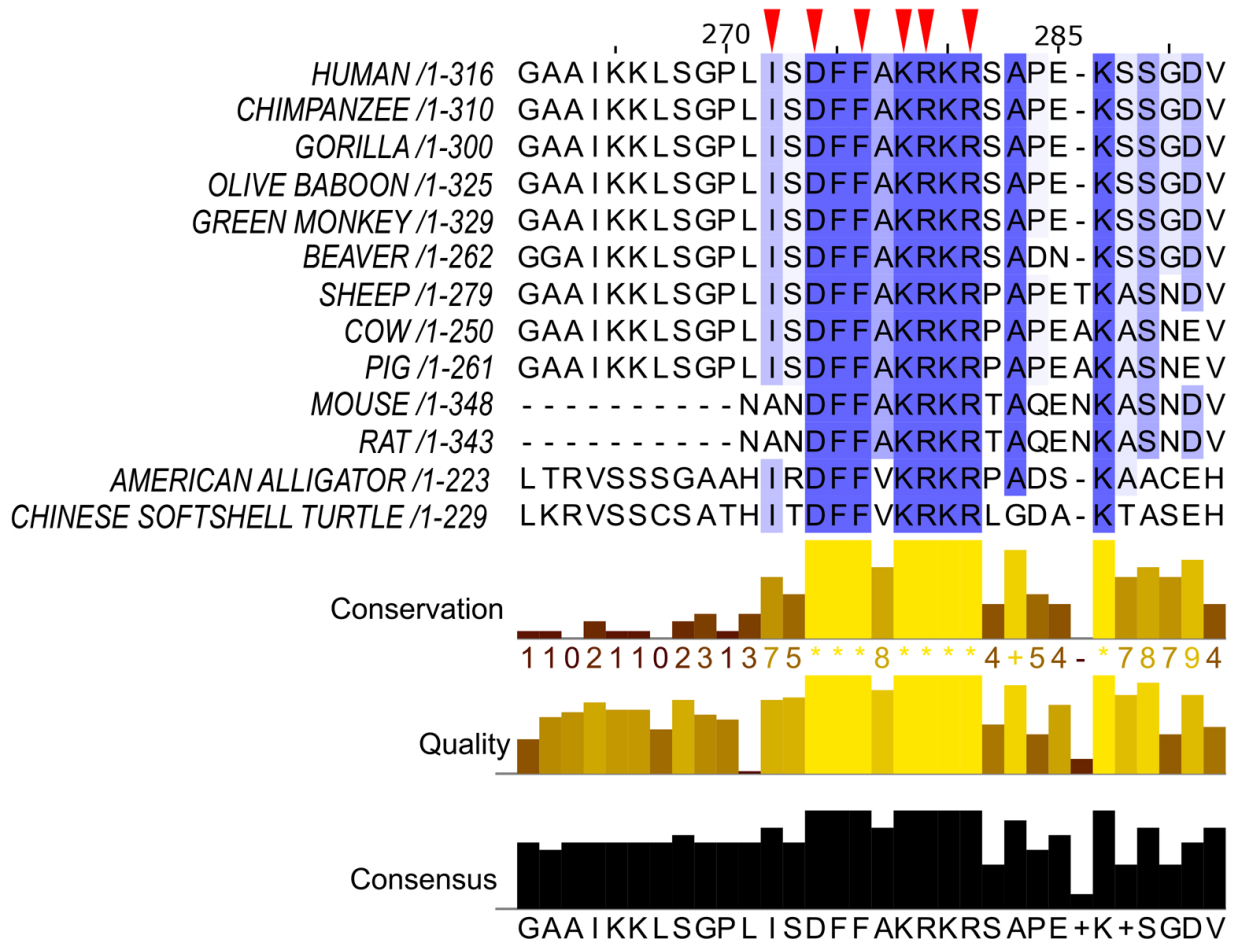
Amino-acid conservancy in the “hot-spot” region of CDKN1C. Red arrowheads represent codons that are mutated in IMAGe syndrome, FGR/IUGR or Silver-Russell syndrome. Yellow asterisks represent complete conservation amongst the species shown.

Review of available data revealed seven publications describing isolated individuals (7) or families (5) with IMAGe syndrome and adrenal insufficiency, who had pathogenic variants in a key region of the PCNA-binding domain of CDKN1C affecting codons 272, 274, 276, 278 and 279 ( Figure 1, Table 2) ( Arboleda *et al.*, 2012; Bodian *et al.*, 2014; Brioude *et al.*, 2013; Hamajima *et al.*, 2013; Kato *et al.*, 2014; Kerns *et al.*, 2014; Sabir *et al.*, 2019). These codons are highly conserved amongst species ( Figure 2). Multiple individuals from different ancestral backgrounds were found to have p.Asp274Asn, p.Lys278Glu or p.Arg279Leu changes.

**Table 2.** Reported variants in CDKN1C and associated phenotypes.

Nucleotide variant	Protein change	Isolated/Familial	Phenotype	Reference
c.815T>G	p.Ile272Ser	Familial (3)	IMAGe	Hamajima *et al.*, 2013
c.820G>A	p.Asp274Asn	Isolated	IMAGe	Arboleda *et al.*, 2012
c.820G>A	p.Asp274Asn	Isolated	IMAGe	Kato *et al.*, 2014
c.820G>A	p.Asp274Asn	Isolated	IMAGe (Glucocorticoid)	Kato *et al.*, 2014
c.826T>G	p.Phe276Val	Familial (7)	IMAGe	Arboleda *et al.*, 2012
c.827T>C	p.Phe276Ser	Isolated	IMAGe	Arboleda *et al.*, 2012
c.832A>G	p.Lys278Glu	Isolated	IMAGe	Arboleda *et al.*, 2012
c.832A>G	p.Lys278Glu	Isolated	IMAGe (Mineralocorticoid)	Bodian *et al.*, 2014
c.836G>C	p.Arg279Pro	Isolated	IMAGe	Arboleda *et al.*, 2012
c.836G>T	p.Arg279Leu	Familial (9)	Silver-Russell	Brioude *et al.*, 2013
c.836G>T	p.Arg279Leu	Familial (2)	Silver-Russell	Sabir *et al.*, 2019
c.842G>T	p.Arg281Ile	Familial (15)	IUGR, short stature, IGT/DM	Kerns *et al.*, 2014

Abbreviations: DM, diabetes mellitus; IGT, impaired glucose tolerance; IMAGe, intrauterine growth restriction, metaphyseal dysplasia, adrenal hypoplasia congenita, genital anomalies; IUGR, intrauterine growth restriction. Numbers in parentheses refer to the number of affected individuals in each kindred.

Variants in CDKN1C associated with familial Silver-Russell syndrome or growth restriction but normal adrenal function were found towards the carboxyl-terminal region of this “hot-spot” domain (p.Arg279Leu, p.Arg281Ile) ( Figure 1).

Analysis of population data from the gnomAD browser showed a complete absence of variants in the key codons listed. Very rare heterozygous SNVs were found that are predicted to cause p.Ala277Val (11:2905355G>A;1 in 10512 alleles) and p.Ala283Val (11:2905337G>A; rs776541692; 1 in 1158 alleles) changes. Of note, these codons are two of the lesser-conserved amino acids within this “hot-spot” region ( Figure 2).

### Expression of CDKN1C in human tissue

**Figure 3.**
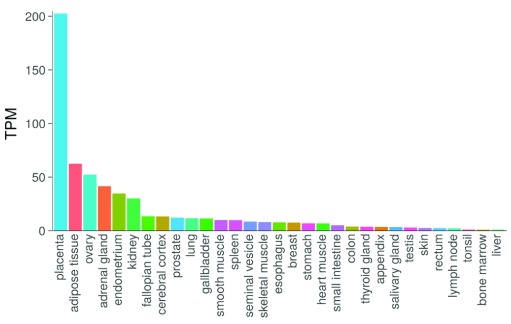
RNA expression of CDKN1C in placenta and different adult human tissues. Data reproduced and modified with permission from the Human Protein Atlas ( www.proteinatlas.org) ( Uhlen *et al.*, 2015). TPM = Transcripts Per Million.

RNA-Seq analysis of *CDKN1C* in a panel of human tissues showed highest expression in the placenta ( Figure 3; Human Protein Atlas data, https://www.proteinatlas.org/ENSG00000129757-CDKN1C/tissue), with strong expression also in adipose tissue, ovary, adrenal, endometrium and kidney. Immunohistochemistry in the Human Protein Atlas repository shows strong staining in the nuclei of both decidual and trophoblastic cells ( https://www.proteinatlas.org/ENSG00000129757-CDKN1C/tissue/placenta).

### Analysis of CDKN1C variants in growth restriction

Analysis of the PCNA-binding domain of *CDKN1C* by Sanger sequencing in a cohort of 58 children with isolated (non-familial) Silver-Russell syndrome did not reveal any pathogenic variants. Sequencing data for exon 2 is shown in Supplementary Data 1 ( Achermann, 2018).

A next-generation sequencing (NGS) approach of *CDKN1C* in children with SRS (n=8), IUGR/FGR (n=37), products of conception (n=22), and mothers with a history of recurrent miscarriage (n=78) also did not reveal pathogenic variants in this PCNA-binding domain region ( Table 1).

Details of coverage for each sample/cohort and each nucleotide is shown in Supplementary Data 2–8 ( Achermann, 2018).

### Analysis of CDKN1C variants outside of the PCNA Binding Domain 

Extended analysis of other regions of *CDKN1C* did not reveal any nonsense, frameshift and canonical splice site variants, which is not surprising as these variants would be loss of function and would be predicted to be associated with overgrowth.

We did identify two heterozygous, non-synonymous missense variants in the cohort of 78 women with recurrent miscarriages. One of these is a 9:g.2906703A>G variant (rs201715947) predicted to result in a p.Leu6Pro change that is present in gnomAD with allelic frequency of 0.0001102 (8/72604). The other variant is a 9:g.2906589C>T change predicted to result in p.Arg44His. This variant is not present in gnomAD but a p.Arg44Leu is present in one individual. We cannot definitively say whether these variants are clinically relevant and pathogenic, but feel it is unlikely.

## Discussion

CDKN1C is now well-established as a key regulator of cell cycle and growth through G1 phase cell cycle arrest. Although loss-of-function of CDKN1C is known to cause macrosomia as part of Beckwith-Wiedemann Syndrome, it is only in the past eight years that “gain-of-function” variants in CDKN1C have been shown to cause growth restriction and IMAGe syndrome. These findings demonstrate clearly how opposite effects in protein function can have opposite phenotypes ( Arboleda *et al.*, 2012; Eggermann *et al.*, 2014). Sometimes these features affect not just growth but also endocrine systems (e.g. adrenal tumours (BWS)/adrenal hypoplasia (IMAGe); congenital hyperinsulinism (BWS)/diabetes mellitus (one family with growth restriction)).

Review of published literature confirms that *CDKN1C* SNVs associated with IMAGe syndrome or growth restriction are all located within a “hot-spot” of the PCNA-binding domain of the protein. The exact function of this region is not clear, and the crystal structure of this region of CDKN1C has not yet been solved. Studies to date suggest pathogenic variants may increase protein stability or reduce the rate of degradation, thereby enhancing the negative effects of CDKN1C on cell cycle progression ( Borges *et al.*, 2015; Hamajima *et al.*, 2013). The very localised nature of these variants clearly demonstrates a key role for this region in CDKN1C function.

*CDKN1C* is strongly expressed in fetal adrenal development as shown by qPCR and immunohistochemistry ( Arboleda *et al.*, 2012), by microarray ( Del Valle *et al.*, 2017), and RNA-Seq (unpublished). Furthermore, analysis of RNA-Seq data from the Human Protein Atlas shows marked expression in the placenta ( Figure 3). Immunohistochemistry shows strong nuclear staining in both decidual and trophoblastic cells. Therefore, gain-of-function of CDKN1C in the developing placenta could have a significant contribution to the growth restriction phenotype.

Further evidence for a potential role for *CDKN1C* in fetal growth restriction phenotypes has emerged with reports of *CDKN1C* variants in familial growth restriction and familial SRS ( Brioude *et al.*, 2013; Kerns *et al.*, 2014; Sabir *et al.*, 2019). Several features of IMAGe syndrome and SRS overlap, such as bi-frontal bossing and relative micrognathia. To date these children have not shown evidence of adrenal insufficiency. Whilst a lack of adrenal features could be due to underlying mosaicism or a somatic “rescue” event, as recently reported for variants in SAMD9 in the related condition MIRAGE syndrome ( Buonocore *et al.*, 2017; Narumi *et al.*, 2016), the fact that several individuals in a family with the same *CDKN1C* SNV were affected provides strong evidence that the primary genomic event is influencing the phenotype rather than a rescue mechanism. Of note, review of the two SNVs associated with FGR and normal adrenal function reveals that they affect amino acids at the C-terminal region of the hotspot. In two families a charged arginine at codon 279 is replaced by a non-polar leucine, whereas a proline at this position is found in classic IMAGe syndrome. In another family, the arginine at position 281 is replaced by a non-polar isoleucine. In some situations, variants in amino-acids flanking critical motifs can be associated with milder phenotypes ( Kyriakakis *et al.*, 2017).

Despite these findings, we did not identify any *CDKN1C* variants in children with isolated (non-familial) SRS or IUGR/FGR. These results are similar to data from Brioude *et al*. who only reported a *CDKN1C* variant in familial SRS and did not find variants in 68 children with an isolated condition ( Brioude *et al.*, 2013). Whilst this does not exclude *CDKN1C* as a potential cause of isolated or sporadic SRS/FGR, it does suggest that it is not a common cause.

Identification of such strong expression of *CDKN1C* in the placenta lead us to consider whether gain-of-function/increased stability of CDKN1C could be associated with loss of pregnancy or recurrent miscarriage. This hypothesis was supported further by recent studies linking placental genes to pregnancy loss ( Perez-Garcia *et al.*, 2018); a potential role for placental *CDKN1C* expression in fetal growth and regulation by oestrogen ( Chen *et al.*, 2018; Gou *et al.*, 2017; López-Abad *et al.*, 2016; Unek *et al.*, 2014); and the fact that paternal imprinting of the gene means that a pool of deleterious variants could be present in the population and cause pregnancy loss (together with live births) in women who carry this variant and inherit it from their father.

Analysis was therefore undertaken in a cohort of mothers who had recurrent miscarriages (often together with live birth(s)) and also from products of conception. However, no variants were found in the PCNA binding domain of CDKN1C in these cohorts.

This work has several limitations. Although 203 total individuals were studied, each sub group is still relatively small and rare *CDKN1C* variants might be discovered if the sample size is increased. The causes of recurrent miscarriage and fetal growth restriction are clearly diverse and can be influenced by many factors, and regulatory regions or enhancers ( Perez-Garcia *et al.*, 2018; Rai & Regan, 2006; Stalman *et al.*, 2018; Wakeling *et al.*, 2017). Nevertheless, this study does highlight the role of the key region of *CDKN1C* in human fetal growth restriction phenotypes, and starts to address the potential role of single gene growth restriction phenotypes in more common obstetric and fetal conditions.

## Data availability

Data has been uploaded to OSF: http://doi.org/10.17605/OSF.IO/Y7KZV ( Achermann, 2018). Representative sequencing data for chromatograms is shown in Supplementary Data 1. Data of coverage for next generation sequencing is shown in Supplementary Data 2–8.

Data are available under the terms of the Creative Commons Zero "No rights reserved" data waiver (CC0 1.0 Public domain dedication).

Access for samples from the Baby Bio Bank is available and can be requested from the steering committee by emailing the Baby Bio Bank Manager ( nita.solanky@ucl.ac.uk). More information about data access can be found on the website under the ‘Protocol for the management of the Baby Bio Bank’, section 15: https://www.ucl.ac.uk/child-health/research/genetics-and-genomic-medicine-programme/baby-biobank. An application form for the use of Baby Bio Bank resources must be completed prior to application (see appendix 2 in the protocol).

## References

[ref-1] Achermann J: Analysis of CDKN1C in fetal growth restriction and pregnancy loss. *Open Science Framework.* 2018 10.17605/OSF.IO/Y7KZV PMC6713069 31497289

[ref-2] Arboleda VA Lee H Parnaik R: Mutations in the PCNA-binding domain of *CDKN1C* cause IMAGe syndrome. *Nat Genet.* 2012;44(7):788–792. 10.1038/ng.2275 22634751 PMC3386373

[ref-3] Bennett J Schrier Vergano SA Deardorff MA: IMAGe syndrome. *GeneReviews®*.1993; (Accessed: 25 April 2018).

[ref-4] Bodian DL Solomon BD Khromykh A: Diagnosis of an imprinted-gene syndrome by a novel bioinformatics analysis of whole-genome sequences from a family trio. *Mol Genet Genomic Med.* 2014;2(6):530–538. 10.1002/mgg3.107 25614875 PMC4303223

[ref-5] Bonaldi A Mazzeu JF Costa SS: Microduplication of the ICR2 domain at chromosome 11p15 and familial Silver-Russell syndrome. *Am J Med Genet A.*Wiley-Blackwell,2011;155A(10):2479–2483. 10.1002/ajmg.a.34023 21910219

[ref-6] Boonen SE Freschi A Christensen R: Two maternal duplications involving the *CDKN1C* gene are associated with contrasting growth phenotypes. *Clin Epigenetics.* 2016;8(1):69. 10.1186/s13148-016-0236-z 27313795 PMC4910218

[ref-7] Borges KS Arboleda VA Vilain E: Mutations in the PCNA-binding site of CDKN1C inhibit cell proliferation by impairing the entry into S phase. *Cell Div.* 2015;10(1):2. 10.1186/s13008-015-0008-8 25861374 PMC4389716

[ref-8] Brioude F Oliver-Petit I Blaise A: *CDKN1C* mutation affecting the PCNA-binding domain as a cause of familial Russell Silver syndrome. *J Med Genet.* 2013;50(12):823–830. 10.1136/jmedgenet-2013-101691 24065356

[ref-35] Buonocore F Clifford-Mobley O King TFJ: Next-generation sequencing reveals novel genetic variants (SRY, DMRT1, NR5A1, DHH, DHX37) in adults with 46,XY DSD. *J Endocr Soc.* 2019;3(12):2341–2360. 10.1210/js.2019-00306 31745530 PMC6855215

[ref-9] Buonocore F Kühnen P Suntharalingham JP: Somatic mutations and progressive monosomy modify *SAMD9*-related phenotypes in humans. *J Clin Invest.* 2017;127(5):1700–1713. 10.1172/JCI91913 28346228 PMC5409795

[ref-10] Cabrera-Salcedo C Kumar P Hwa V: IMAGe and related undergrowth syndromes: the complex spectrum of gain-of-function CDKN1C mutations. *Pediatr Endocrinol Rev.* 2017;14(3):289–297. 28508599 10.17458/per.vol14.2017.SKHD.imageandrelatedundergrowth

[ref-11] Chen XJ Chen F Lv PP: Maternal high estradiol exposure alters CDKN1C and IGF2 expression in human placenta. *Placenta.* 2018;61:72–79. 10.1016/j.placenta.2017.11.009 29277274

[ref-31] Del Valle I Buonocore F Duncan AJ: A genomic atlas of human adrenal and gonad development [version 2; referees: 4 approved]. *Wellcome Open Res.* 2017;2:25. 10.12688/wellcomeopenres.11253.2 28459107 PMC5407452

[ref-12] Eggermann T Binder G Brioude F: *CDKN1C* mutations: two sides of the same coin. *Trends Mol Med.* 2014;20(11):614–622. 10.1016/j.molmed.2014.09.001 25262539

[ref-13] Gou C Liu X Shi X: Placental expressions of *CDKN1C* and *KCNQ1OT1* in monozygotic twins with selective intrauterine growth restriction. *Twin Res Hum Genet.* 2017;20(5):389–394. 10.1017/thg.2017.41 28803575

[ref-14] Guran T Buonocore F Saka N: Rare causes of primary adrenal insufficiency: genetic and clinical characterization of a large nationwide cohort. *J Clin Endocrinol Metab.* 2016;101(1):284–292. 10.1210/jc.2015-3250 26523528 PMC4701852

[ref-15] Hamajima N Johmura Y Suzuki S: Increased protein stability of CDKN1C causes a gain-of-function phenotype in patients with IMAGe syndrome. *PLoS One.*Edited by He B,2013;8(9):e75137. 10.1371/journal.pone.0075137 24098681 PMC3787065

[ref-16] Kato F Hamajima T Hasegawa T: IMAGe syndrome: clinical and genetic implications based on investigations in three Japanese patients. *Clin Endocrinol (Oxf).* 2014;80(5):706–713. 10.1111/cen.12379 24313804

[ref-17] Kerns SL Guevara-Aguirre J Andrew S: A novel variant in *CDKN1C* is associated with intrauterine growth restriction, short stature, and early-adulthood-onset diabetes. *J Clin Endocrinol Metab.* 2014;99(10):E2117–E2122. 10.1210/jc.2014-1949 25057881 PMC4184067

[ref-18] Kyriakakis N Shonibare T Kyaw-Tun J: Late-onset X-linked adrenal hypoplasia (DAX-1, *NR0B1*): two new adult-onset cases from a single center. *Pituitary.* 2017;20(5):585–593. 10.1007/s11102-017-0822-x 28741070 PMC5606946

[ref-19] Lek M Karczewski KJ Minikel EV: Analysis of protein-coding genetic variation in 60,706 humans. *Nature.*Nature Publishing Group,2016;536(7616):285–291. 10.1038/nature19057 27535533 PMC5018207

[ref-20] Leon LJ Solanky N Stalman SE: A new biological and clinical resource for research into pregnancy complications: The Baby Bio Bank. *Placenta.* 2016;46:31–37. 10.1016/j.placenta.2016.08.085 27697219 PMC5062948

[ref-21] López-Abad M Iglesias-Platas I Monk D: Epigenetic characterization of *CDKN1C* in placenta samples from non-syndromic intrauterine growth restriction. *Front Genet.* 2016;7:62. 10.3389/fgene.2016.00062 27200075 PMC4844605

[ref-23] Narumi S Amano N Ishii T: *SAMD9* mutations cause a novel multisystem disorder, MIRAGE syndrome, and are associated with loss of chromosome 7. *Nat Genet.* 2016;48(7):792–797. 10.1038/ng.3569 27182967

[ref-24] Perez-Garcia V Fineberg E Wilson R: Placentation defects are highly prevalent in embryonic lethal mouse mutants. *Nature.* 2018;555(7697):463–468. 10.1038/nature26002 29539633 PMC5866719

[ref-25] Rai R Regan L: Recurrent miscarriage. *Lancet.* 2006;368(9535):601–11. 10.1016/S0140-6736(06)69204-0 16905025

[ref-36] Sabir AH Ryan G Mohammed Z: Familial Russell-Silver syndrome like phenotype in the PCNA domain of the *CDKN1C* gene, a further case. *Case Rep Genet.* 2019;1398250. 10.1155/2019/1398250 31976094 PMC6959155

[ref-26] Schönherr N Meyer E Roos A: The centromeric 11p15 imprinting centre is also involved in Silver-Russell syndrome. *J Med Genet.*BMJ Publishing Group Ltd,2007;44(1):59–63. 10.1136/jmg.2006.044370 16963484 PMC2597902

[ref-27] Stalman SE Solanky N Ishida M: Genetic analyses in small-for-gestational-age newborns. *J Clin Endocrinol Metab.* 2018;103(3):917–925. 10.1210/jc.2017-01843 29342293

[ref-28] Stampone E Caldarelli I Zullo A: Genetic and epigenetic control of *CDKN1C* expression: importance in cell commitment and differentiation, tissue homeostasis and human diseases. *Int J Mol Sci.* 2018;19(4): pii: E1055. 10.3390/ijms19041055 29614816 PMC5979523

[ref-29] Uhlen M Fagerberg L Hallström BM: Proteomics. Tissue-based map of the human proteome. *Science.* 2015;347(6220):1260419. 10.1126/science.1260419 25613900

[ref-30] Unek G Ozmen A Ozekinci M: Immunolocalization of cell cycle proteins (p57, p27, cyclin D3, PCNA and Ki67) in intrauterine growth retardation (IUGR) and normal human term placentas. *Acta Histochemica.* 2014;116(3):493–502. 10.1016/j.acthis.2013.10.007 24252562

[ref-32] Vilain E Le Merrer M Lecointre C: IMAGe, a new clinical association of intrauterine growth retardation, metaphyseal dysplasia, adrenal hypoplasia congenita, and genital anomalies. *J Clin Endocrinol Metab.* 1999;84(12):4335–4340. 10.1210/jcem.84.12.6186 10599684

[ref-33] Wakeling EL Brioude F Lokulo-Sodipe O: Diagnosis and management of Silver-Russell syndrome: first international consensus statement. *Nat Rev Endocrinol.* 2017;13(2):105–124. 10.1038/nrendo.2016.138 27585961

[ref-34] Waterhouse AM Procter JB Martin DM: Jalview Version 2--a multiple sequence alignment editor and analysis workbench. *Bioinformatics.*Oxford University Press,2009;25(9):1189–1191. 10.1093/bioinformatics/btp033 19151095 PMC2672624

