# Supplementary material for: Analysis of CDKN1C in fetal growth restriction and pregnancy loss
Source: F1000Res. 2020 Apr 21;8:90. Originally published 2019 Jan 23. [Version 2] doi: 10.12688/f1000research.15016.2 (PMC6713069; doi:10.12688/f1000research.15016.2)
Supplement: Peer review history

# Peer review: Reviewer response for version 2

**DOI:** 10.5256/f1000research.25093.r62553

**Published:** 2020-04-22

**Authors:** Amanda J. Drake, Kahyee Hor

**Affiliations:** 1 University/British Heart Foundation Centre for Cardiovascular Science, The Queen’s Medical Research Institute, University of Edinburgh, Edinburgh, UK; 2 University/British Heart Foundation Centre for Cardiovascular Science, The Queen’s Medical Research Institute, University of Edinburgh, Endinburgh, UK

Approved

Is the work clearly and accurately presented and does it cite the current literature?

Yes

If applicable, is the statistical analysis and its interpretation appropriate?

Yes

Are all the source data underlying the results available to ensure full reproducibility?

Yes

Is the study design appropriate and is the work technically sound?

Yes

Are the conclusions drawn adequately supported by the results?

Yes

Are sufficient details of methods and analysis provided to allow replication by others?

Yes

Reviewer Expertise:

Epigenetics, paediatrics, fetal growth, obstetrics

We confirm that we have read this submission and believe that we have an appropriate level of expertise to confirm that it is of an acceptable scientific standard.

# Peer review: Reviewer response for version 1

**DOI:** 10.5256/f1000research.16349.r51957

**Published:** 2019-08-27

**Authors:** Amanda J. Drake, Kahyee Hor

**Affiliations:** 1 University/British Heart Foundation Centre for Cardiovascular Science, The Queen’s Medical Research Institute, University of Edinburgh, Edinburgh, UK; 2 University/British Heart Foundation Centre for Cardiovascular Science, The Queen’s Medical Research Institute, University of Edinburgh, Endinburgh, UK

In this report Suntharalingham and colleagues investigate the extent to which variations in a known negative regulator of cell growth (CDKN1C) may be involved in isolated growth restriction or recurrent pregnancy loss. 

 They approach this in several ways:

1. Through analysis of published literature
2. Through analysis of CDKN1C expression in multiple tissues using available RNA-seq data
3. Targeted sequencing in a number of patient populations.

As a result of the literature search, they conclude that reported variants are located in the PCNA-binding domain of CDKN1C and therefore focus on this for their sequencing analysis. The analysis of available RNA-seq data suggests that the gene is highly expressed in placenta, suggesting it may have a role in pregnancy maintenance. Sequencing analysis revealed no pathogenic variants in the targeted region of CDKN1C suggesting this is an uncommon cause of fetal growth restriction or recurrent pregnancy loss.

 The paper is well written and easy to read, with appropriate citation of the literature.

 Minor comments:

1. PubMed search terms appear quite limited. Are the authors certain they have not missed any papers by not  including terms such as IUGR, FGR, SGA?
2. Do the pregnancies/children in the FGR/IUGR group have any other complications?
3. The term 'usually a history of live births' in table 2 is rather vague. Were any of these live births also affected by FGR/IUGR etc. or by pregnancy complications commonly associated with poor fetal growth e.g. pre-eclampsia.
4. In table 2 - do the numbers in brackets refer to the number of families with that variant or the number of affected individuals within a family?

Given the limited number of publications describing pathogenic mutations in the PCNA-binding domain of CDKN1C in IMAGe syndrome, familial SRS and in a single family with FGR and early adult onset diabetes, are the researchers planning to analyse other regions of the gene/its regulatory regions for other variations which may affect expression? Could they take this on to include analysis of placental gene expression/protein expression/function in assocision with analysis of sequence variation?

Is the work clearly and accurately presented and does it cite the current literature?

Yes

If applicable, is the statistical analysis and its interpretation appropriate?

Yes

Are all the source data underlying the results available to ensure full reproducibility?

Yes

Is the study design appropriate and is the work technically sound?

Yes

Are the conclusions drawn adequately supported by the results?

Yes

Are sufficient details of methods and analysis provided to allow replication by others?

Yes

Reviewer Expertise:

Epigenetics, paediatrics, fetal growth, obstetrics

We confirm that we have read this submission and believe that we have an appropriate level of expertise to confirm that it is of an acceptable scientific standard.

# Response

**Published:** 2020-04-08

**Authors:** John Achermann

**Affiliations:** UCL Great Ormond Street Institute of Child Health, UK

*Minor comments:*

*1. PubMed search terms appear quite limited. Are the authors certain they have not missed any papers by not including terms such as IUGR, FGR, SGA?*

Response: Thank you for this comment. We have used those additional search terms and not found any additional publications. We have modified the methods accordingly. A more recent search of CDKN1C and IMAGe has revealed an interesting additional family of two half-siblings with Silver Russell Syndrome (SRS) (Sabir et al 2019), who have a c.836 G> T variant resulting in the p.Arg279Leu change previously reported by Brioude et al. We have modified Figure 1 and Table 2 to include these important new data, which supports the hypothesis that the p.Arg279Leu change (at the edge of the PCNA-binding domain) is associated with SRS, rather than the more severe IMAGe phenotype. We have added this new reference to the paper and clarified the footnote in Table 2 to show that the numbers in parentheses refer to numbers of affected individuals within each kindred.

*2. Do the pregnancies/children in the FGR/IUGR group have any other complications?*

Response: To our knowledge this group do not have additional multisystem features.

*3. The term 'usually a history of live births' in table 2 is rather vague. Were any of these live births also affected by FGR/IUGR etc. or by pregnancy complications commonly associated with poor fetal growth e.g. pre-eclampsia.*

Response: This is a good point. Unfortunately, we do not have easy access to those data at present.

*4. In table 2 - do the numbers in brackets refer to the number of families with that variant or the number of affected individuals within a family?*

Response: Thank you for pointing this out. he number refers to the number of affected individuals within a family.  We have added a footnote to clarify this. 

*Given the limited number of publications describing pathogenic mutations in the PCNA-binding domain of CDKN1C in IMAGe syndrome, familial SRS and in a single family with FGR and early adult onset diabetes, are the researchers planning to analyse other regions of the gene/its regulatory regions for other variations which may affect expression? Could they take this on to include analysis of placental gene expression/protein expression/function in association with analysis of sequence variation?*

Response: We have analysed the rest of CDKN1C beyond the PCNA-binding domain wherever possible and did not find any variants that are clearly pathogenic (see response to reviewer 1). We did not build promoter or regulatory regions of *CDKN1C* into our capture design but it is an interesting point and maybe this can be addressed as more whole genome sequencing data become available. As pointed out, correlating genomic variations with placental gene and protein expression in the same individual would be a very powerful approach, if appropriate samples were available.

# Peer review: Reviewer response for version 1

**DOI:** 10.5256/f1000research.16349.r50625

**Published:** 2019-07-18

**Authors:** Flavia Cerrato

**Affiliations:** 1 Department of Environmental, Biological and Pharmaceutical Sciences and Technologies, University of Campania Luigi Vanvitelli, Caserta, Italy

In this paper Suntharalingham and coworkers investigate the involvement of CDKN1C, a negative regulator of cell proliferation and growth, in fetal growth restriction (FGR)/intrauterine growth restriction (IUGR) human conditions, pregnancy loss or recurrent miscarriage. While many loss-of-function variants have been reported associated with the overgrowth condition Beckwith-Wiedemann syndrome and affecting all the domains of the protein, only few variants predicted to be “gain of function”, have been found associated with syndromic growth restriction conditions (IMAGe and SRS). The authors review the latter cases and find that all reported variants, associated with growth restriction, are localized in the same region inside the PCNA-binding domain, and affect evolutionary conserved amino acids. 

 By studying RNA-seq data available on the Human Protein Atlas website, the authors consider the high expression level of *CDKN1C *in placenta to hypothesize its involvement in pregnancy loss and miscarriage as a consequence of placenta restriction. Following a DNA targeted sequencing analysis restricted to the “hot spot” PCNA-binding domain in isolated cases of SRS (n=58), IUGR/FGR (n=26), loss of pregnancy (n=21) and women with recurrent miscarriage (n=71), they do not find any pathogenic variant, concluding that pathogenic variants of this analysed region are not a common cause of these pathological conditions.

 The paper is interesting and easy to read. The potential role of CDKN1C in pregnancy maintenance and its putative involvement in pregnancy loss is interesting, and an aspect that in future could be better investigated by extending the number of the cases to analyze. 

 It would be interesting if the authors could report the analysis of the variants extended to the CDKN1C domains other than the region corresponding to the “hot spot” PCNA-binding domain (at least for cases analysed by NGS). 

 Minor comments:

- The authors should clarify why they have analyzed *CDKN1C *variants only in the isolated SRS cases, excluding the familial cases.
- Most of information reported in Table 2 are already present in Figure 1, therefore Table 2 could be removed or reported as Supplementary Table.

Is the work clearly and accurately presented and does it cite the current literature?

Yes

If applicable, is the statistical analysis and its interpretation appropriate?

Yes

Are all the source data underlying the results available to ensure full reproducibility?

Yes

Is the study design appropriate and is the work technically sound?

Yes

Are the conclusions drawn adequately supported by the results?

Yes

Are sufficient details of methods and analysis provided to allow replication by others?

Yes

Reviewer Expertise:

Genomic imprinting, Imprinting disorders

I confirm that I have read this submission and believe that I have an appropriate level of expertise to confirm that it is of an acceptable scientific standard.

# Response

**Published:** 2020-04-08

**Authors:** John Achermann

**Affiliations:** UCL Great Ormond Street Institute of Child Health, UK

*The paper is interesting and easy to read. The potential role of CDKN1C in pregnancy maintenance and its putative involvement in pregnancy loss is interesting, and an aspect that in future could be better investigated by extending the number of the cases to analyze.*

Response: In the revised submission, we have used the Agilent HaloPlex panel, as well as a Nonacus 3T cell target enrichment approach to add data from an additional 27 subjects (POC (n= 1); women with history of recurrent miscarriage (n=7); Silver Russell Syndrome (n=8) and fetal growth restriction (n=11) ). We have modified Table 1, Supplementary data and Figures, and relevant places in the text, accordingly, and included a Supplementary Data 8 for Nonacus coverage. A reference for the Nonacus methods in included (Buonocore et al., 2019). 

*It would be interesting if the authors could report the analysis of the variants extended to the CDKN1C domains other than the region corresponding to the “hot spot” PCNA-binding domain (at least for cases analysed by NGS).*

Response: Although our focus was on the PCNA-binding domain, we agree it is valid to look at regions outside of the hotspot and thank the reviewer (and reviewer 2) for the suggestion.

Analysis of CDKN1C using the gnomAD browser (accessed March 2020) shows an expected number of synonymous changes (o/e =1.06 (0.86-1.31)) but fewer missense (o/e= 0.51 (0.42-0.63)) and pLoF changes (o/e=0 (0-0.53)) than expected. ( https://gnomad.broadinstitute.org/gene/ENSG00000129757?dataset=gnomad_r2_1)

Based on the fact that random variants within a gene are more likely to cause disruption rather than gain-of-function, the low number of observed missense changes is more likely to represent an intolerance to disruption and loss of function associated with overgrowth and Beckwith-Wiedemann Syndrome, rather than rare gain of function changes associated with FGR/IMAGe syndrome. 

We have undertaken an extended analysis of data for the gene. We did not detect any category 1 variants (nonsense, frameshift, canonical splice site variants) at all, which is not surprising, as these would be loss of function and predicted to be associated with overgrowth.

We only identified two category 2 (non-synonymous missense) variants in the cohort, both in women who have had recurrent miscarriages.  One of these is a 9:g.2906703A>G variant (rs201715947) predicted to result in a p.Leu6Pro change that is present in gnomAD with allelic frequency of 0.0001102 (8/72604).  The other variant is a 9:g.2906589C>T predicted to result in a p. Arg44His. This variant is not present in gnomAD but a p.Arg44Leu is present in one male. Although the SIFT score for this variant suggests it is deleterious, we find that prediction models are not always reliable for gain-of-function effects. On balance, we do not feel that either of these variants are likely to be clinically pathogenic although we cannot say this definitively.  We have commented on this in the revised results. More certain is our finding that there is not a large pool of additional CDKN1C variants in our cohort outside of the PCNA binding domain.

*Minor comments:*

*•  The authors should clarify why they have analyzed CDKN1C variants only in the isolated SRS cases, excluding the familial cases.*

Response: Our cohort was only made up of sporadic Silver Russell Syndrome subjects; we had no familial cases. We have mentioned this in the methods. 

*•  Most of information reported in Table 2 are already present in Figure 1, therefore Table 2 could be removed or reported as Supplementary Table 2.*

Response: We agree there is some overlap, but we would prefer to keep both in the main body of text as Figure 1 shows the structure of CDKN1C and position of variants, and Table 2 shows nucleotide data, whether mineralocorticoid or glucocorticoid was given, and links to the original references.
